# Chromatin accessibility of the jejunum in two high-yielding laying hen strains under divergent mineral phosphorus supply during the transition to egg laying

**DOI:** 10.1016/j.aninu.2026.01.015

**Published:** 2026-06-08

**Authors:** Yosef A. Abitew, Henry Reyer, Frieder Hadlich, Michael Oster, Nares Trakooljul, Vera Sommerfeld, Markus Rodehutscord, Klaus Wimmers, Siriluck Ponsuksili

**Affiliations:** a Research Institute for Farm Animal Biology (FBN), Dummerstorf 18196, Mecklenburg-Vorpommern, Germany; b Institute of Animal Science, University of Hohenheim, Stuttgart 70599, Baden-Württemberg, Germany; c Faculty for Agriculture, Civil and Environmental Engineering, University of Rostock, Rostock 18059, Mecklenburg-Vorpommern, Germany

**Keywords:** Phosphorus, Chromatin accessibility, Gene expression, Laying hen, Mineral metabolism

## Abstract

Optimizing dietary phosphorus (P) levels is crucial for sustainable egg production, hen welfare, productivity, and reducing excessive P excretion. Dietary P influences systemic physiological responses, including gene expression dynamics and epigenetic regulation, particularly in the gut. Epigenetic modifications, including chromatin accessibility, play a crucial role in gene regulation by integrating genetic and environmental signals. It is hypothesized that age, strain, and mineral P levels together influence the epigenetic and transcriptional landscape in the jejunum during the transition to egg-laying. Using the domestic chicken (*Gallus gallus domesticus*), two commercial laying-hen strains, Lohmann Brown (LB) and Lohmann Selected Leghorn (LSL) were studied. The experiment followed a 2 × 2 × 2 factorial design including two strains (LB vs. LSL), two production periods (transition before the onset of egg laying vs. onset of laying), and two dietary P levels (without supplemental mineral P [P−] or with 1 g/kg supplemental P from monocalcium phosphate [P+]). A total of 80 hens were used, 10 per strain × period × diet combination, in a randomised complete block design. In each period, hens were housed individually in metabolic units for 4 weeks, beginning either before the onset of egg-laying (transition; placed at 15 weeks of age, sampled at week 19) or after the onset of egg-laying (onset of laying; placed at 20 weeks of age, sampled at week 24). At placement, mean initial body weight was 1228 ± 134 g (LB) and 1036 ± 70 g (LSL) in the transition period, and 1552 ± 93 g (LB) and 1244 ± 64 g (LSL) in the onset-of-laying period. Chromatin accessibility was assessed using an assay for transposase-accessible chromatin with sequencing (ATAC-Seq). The analysis revealed distinct strain-specific chromatin accessibility profiles, identifying 6610 differentially accessible regions (DAR) (adjusted *P* < 0.01). The LSL strain genome exhibited more regions with reduced accessibility (3603 DAR) compared to the LB strain (adjusted *P* < 0.01). Extensive chromatin remodelling was observed during the transition to laying onset (7884 DARs), with greater loss of accessibility at 24 weeks (4193 DARs). Dietary P had subtler effects (1671 DARs; *P* < 0.01), including increased accessibility at loci such as *CALB1* under the P- diet. Enrichment analysis of transcripts nearest these DARs revealed pathways related to lipid transport, fatty acid metabolism, Wnt signalling, transforming growth factor-β (TGF-β) signalling, and calcium signalling. Additionally, ANalysis Algorithm for Networks Specified by Enhancers (ANANSE) integrates predicted transcription factor (TF) binding in accessible chromatin with RNA-Seq expression to weight gene regulatory networks (GRN) edges and rank TF influence (P+ vs. P-), highlighting regulators linked to bone/cartilage remodelling (*RUNX2*, *STAT3*, and *CLOCK*) and stress signalling (*ATF3* and *JUND*). This study elucidated the complex interplay between sexual maturation period, genetic background, and nutritional mineral status in shaping the regulatory architecture of the jejunum, a key site for P absorption.

## 1 Introduction

Improving phosphorus (P) utilization efficiency is of high practical importance, as P supplementation represents a major cost in layer diets and excessive P excretion contributes to environmental pollution and global P depletion (Pongmanee et al., 2020; Rodehutscord et al., 2023). A deeper understanding of the genetic and epigenetic mechanisms shaping P utilization can therefore support precision nutrition strategies that maintain egg production and shell quality while reducing feed costs and the ecological footprint of poultry farming. Epigenetic modifications, such as DNA methylation, histone modifications, and chromatin accessibility, play essential roles in integrating genetic and environmental signals, adding a layer of complexity to the understanding of gene regulation as reviewed previously (Gibney and Nolan, 2010). The assay for transposase-accessible chromatin using sequencing (ATAC-Seq) is an efficient method for investigating the accessibility of chromatin throughout the genome (Buenrostro et al., 2015). This approach enables identification of regulatory elements such as promoters and enhancers by mapping open chromatin regions where transcription factors and other DNA-binding proteins interact with DNA. Previous studies on chicken epigenetics have focused on DNA methylation and post-translational histone modifications, particularly associated with behaviour, nutrient metabolism, ageing and disease resistance, which might influence the performance of chickens (David et al., 2017). Recently, a critical window of epigenetic modulation during the pre-laying period was identified, with stage-specific methylation patterns in the intestinal tissues and higher methylation levels at specific loci in the Lohmann Selected Leghorn (LSL) strain compared to the Lohmann Brown (LB) strain (Ponsuksili et al., 2025).

The functional annotation of animal genomes (FAANG) initiative annotates the transcriptome and chromatin structure of domesticated animals (Foissac et al., 2019). In a recent study, the transcriptional and epigenetic changes during postnatal muscle development of broilers were analysed using RNA-Seq and ATAC-Seq (Gu et al., 2024). A chicken chromatin accessibility atlas of 53 ATAC-Seq samples from 11 tissues has revealed strong tissue specificity of open chromatin, especially in intergenic and intronic regions, implying that gene regulatory elements in these regions may strongly influence tissue-specific gene expression (Zhu et al., 2023). A study examining the chromatin architecture dynamics during folliculogenesis in chicken granulosa cells showed that the dynamic accessibility of chromatin regulates tissue- and age-specific gene expression during poultry reproduction (Li et al., 2022). The role of epigenetic factors, in particular chromatin accessibility, in laying performance and mineral utilization is still largely unexplored.

Because the jejunum is the primary site of P uptake, this segment was selected to capture regulatory changes most relevant to mineral economy at the onset of lay while reducing confounders in chromatin-accessibility measurements. Integrating ATAC-Seq with RNA-Seq will map the gut regulatory landscape underlying health and productivity in laying hens. Given P's central roles in skeletal integrity and eggshell formation, accumulating evidence indicates that intrinsic factors, genetic background and physiological maturation, shape jejunal transcriptomic variation more than external factors such as dietary P content (Ponsuksili et al., 2021; Reyer et al., 2021). The current study builds upon previous work with the same cohort of hens, which established key differences at both the physiological and transcriptomic levels (Abitew et al., 2024; Qasir et al., 2025; Sommerfeld et al., 2024). Lohmann Brown (LB) hens and a diet supplemented with 1 g/kg P from monocalcium phosphate (P+) increased P intake and retention without raising excretion, indicating more efficient mineral P use (Sommerfeld et al., 2024); dietary P supply also significantly affected plasma inorganic P, especially at week 19 under P-deficient diet without mineral P supplement (P-) diets (Qasir et al., 2025). Strain-specific jejunal gene-expression responses to dietary mineral P were further observed (Abitew et al., 2024). Together, the findings implicate jejunal chromatin accessibility as a key epigenetic mechanism of adaptation to low mineral P across ages and strains. ATAC-Seq and RNA-Seq were therefore combined to dissect interactions among strain, age, and P supply, linking chromatin and transcriptomic changes to P-related phenotypes. By profiling the small-intestinal chromatin landscape of two high-yielding hen strains at two developmental stages, with or without monocalcium phosphate supplementation, distinct strain- and age-dependent epigenetic signatures were identified and linked to P-related phenotypes.

## 2 Materials and methods

### 2.1 Animal ethics statement

The experiment was conducted at the agricultural experiment station of the University of Hohenheim, Germany, with the approval of the regional council of Tübingen, approval number HOH67/21 TE, following the German animal welfare legislation.

### 2.2 Experimental design and sample collection

The same samples described previously were used (Abitew et al., 2024; Sommerfeld et al., 2024). Briefly, the study employed a 2 × 2 × 2 factorial design to examine the effects of hen strain (LB and LSL), production period (before vs. after the onset of egg), and dietary P level (P- and P+). A total of 80 domestic laying hens (*Gallus gallus domesticus*) were used in the study, comprising two commercial strains: LB (*n* = 40) and LSL (*n* = 40). Hens were studied in two production periods: a transition period before the onset of egg-laying, in which hens were placed in individual metabolic units at 15 weeks of age and sampled at week 19, and an onset-of-laying period, in which hens were placed at week 20 and sampled at week 24. In each period, hens received either a P- or a P+ diet for 4 weeks. Each of the eight treatment combinations (2 strains × 2 periods × 2 diets) comprised 10 replicates, with one hen per metabolic unit representing one experimental unit; thus 10 hens per group and 80 hens in total, in a randomized complete block design. At placement, mean initial body weight was 1228 ± 134 g (LB) and 1036 ± 70 g (LSL) in the transition period, and 1552 ± 93 g (LB) and 1244 ± 64 g (LSL) in the onset-of-laying period. This dietary contrast resulted in a 1 g/kg difference in non-phytate phosphorus (NPP) between the groups. Forty LB hens and forty LSL hens were raised under standardised conditions. Standard maize-soybean meal diets without supplemental phytase were provided, comprising a developer feed (weeks 15–16), a pre-layer feed (weeks 16–17), and a layer feed (weeks 17-19 and weeks 20–24). Ingredients and nutrient levels of the experimental diets is shown in Table S1 (Sommerfeld et al., 2024). In weeks 15 and 20, ten hens per factor combination were individually housed in metabolism units and provided experimental diets for ad libitum consumption for 4 week before slaughter. Individual hens were weighed at the beginning and end of each 4-week period, on the day of slaughter, and at the start and end of the excreta-collection period. Feed offered and refusals (from troughs and trays) were weighed daily during the experimental period; average daily feed intake (ADFI) was calculated as follows:

ADFI = (Feed offered - Feed refused)/Number of days per hen.

Hens slaughtered at week 19 were given three sequential diets (developer, pre-layer, and layer) P- or P+. The second group followed a similar regimen, receiving the layer feed throughout, until week 24. Total excreta were collected from stainless-steel trays placed underneath each metabolic unit at 24-h intervals for 4 consecutive days. Feathers, skin scales, and spilled feed were removed before each collection, and the excreta were immediately frozen at −20 °C. As previously described (Sommerfeld et al., 2024), excreta samples were thawed at 3 °C, weighed, pooled for each hen and period, and homogenized. A subsample was freeze-dried, pulverized and preserved for subsequent digesta analysis. Feed samples were ground using an Ultra Centrifugal Mill (ZM 200, Retsch GmbH, Haan, North Rhine-Westphalia, Germany) to pass through a 0.5 mm sieve. Dry matter (DM) content was determined in triplicate following method No. 3.1 (VDLUFA, 2007). At the end of each period, hens were stunned and killed, and digesta from the jejunum was collected by gentle squeezing, immediately frozen at −20 °C, freeze-dried, and pulverised (Sommerfeld et al., 2024). Pulverized feed, digesta, and excreta samples were analysed for P, Ca, and Ti by inductively coupled plasma–optical emission spectrometry (ICP-OES) after wet digestion, following a modified method of Boguhn et al. (2009) as detailed by Zeller et al. (2015). InsP_6_ was quantified by high-performance ion chromatography, and analysed NPP was calculated as the difference between analysed total P and phytate-bound P (InsP_6_-P), as detailed in Sommerfeld et al. (2024). Metabolizable energy (ME) was a calculated value obtained during diet formulation from the tabulated nutrient contents of the feed ingredients according to the recommendations of the Gesellschaft für Ernährungsphysiologie (GfE, 1999).

### 2.3 Nuclear extraction, library preparation, and sequencing

The jejunum, the principal site of intestinal P absorption, was selected as the most physiologically relevant tissue to capture regulatory adaptations to dietary P during the transition to lay. Using a fixed 3-cm anterior-jejunal segment across all birds minimized regional variability in epithelial composition and improved between sample comparability for strain, age, and diet effect analyses. The jejunum mucosa of the 80 hens was collected and processed for RNA and nuclear extraction. The ATAC-Seq libraries were generated via an established protocol for cryopreserved nuclei (Buenrostro et al., 2015; Halstead et al., 2020). The samples were collected, slowly frozen, and cryopreserved to maintain the viability of the cells. Density gradient centrifugation with iodexanol was employed to eliminate contaminating mitochondria. Nuclei viability and quantity were assessed via trypan blue staining to ensure ≥ 50,000 intact viable nuclei prior to library construction. Genomic DNA fragmentation was performed with the Illumina Tagment DNA Enzyme and Buffer Kit (Tn5 Transposase/Tagment DNA Enzyme 1; Illumina, Inc., San Diego, CA, USA) following the manufacturer's recommended protocols. Tween 20 and digitonin were added to the transposition reaction mixture to increase nuclear permeabilisation and efficiency. A PCR was performed to amplify transposed fragments using the Illumina unique dual indices, and sequenced for 2 × 100 bp paired-end on a NextSeq 2000 sequencer (Illumina, Inc., San Diego, CA, USA).

### 2.4 ATAC-Seq data processing and differentially accessible peaks

The ATAC-Seq data were processed using the nfcore-atacseq pipeline from NEXTFLOW (Patel et al., 2023). Raw sequencing reads were trimmed to remove adapters and low-quality reads using Trim Galore (version 0.6.7), a wrapper around Cutadapt (version 3.4) and quality assessed with FastQC (version 0.11.9). Trimmed reads were mapped to the chicken genome (Ensembl galGal6, version GCF_000002315.6) assembly via the Burrow-Wheeler Aligner (BWA; version 0.7.17-r1188) with the default parameters. PCR duplicates were marked using Picard's MarkDuplicates option to generate filtered BAM files (version 2.27.4). Reads aligned to the mitochondrial genome were removed from further analysis. Insert sizes were calculated, and BAM files were generated using SAMtools (version 1.16.1). Duplicated reads were removed before peak identification. Owing to its reliability in identifying regions of significant chromatin accessibility, MACS2 (version 2.2.7.1) was used for peak calling with the BAMPE option and the default parameters. The transcription start site (TSS) enrichment score was calculated to assess the quality of the sequencing libraries and ensure a high signal-to-noise ratio. Consensus peaks were identified in all the biological replicates to capture potential batch effects in our analysis.

The R package DESeq2 was employed to determine potential differentially accessible regions (DAR) based on the consensus peaks. To control for potential unwanted variation beyond strain, diet, and age, the svaseq function from the SVA package were applied (v3.46.0). Two surrogate variables (BV1 and BV2) capturing batch effects were estimated from the normalized count matrix and included in the final model together with strain, diet, and age. After the count matrix was transformed to account for library size and compositional differences, the svaseq function was applied to estimate two surrogate variables (BV1 and BV2) that capture potential sources of unwanted variation. These surrogate variables, along with the experimental factors, strain, age, and diet were included in the full model for differential accessibility analysis to identify significant main effects. The following statistical full model was used:

$$y_{\text{ijk}}=\mu +\alpha_{i}+\beta_{j}+\gamma_{k}+BV1+BV2+\varepsilon_{\text{ijk},}$$

where *y*_*ijk*_ is the response vector; *μ* is the overall mean; *α*_*i*_ is the strain effect (fixed); *β*_*j*_ is the effect of dietary P (fixed); *γ*_*k*_ is the age effect (fixed); *BV1* is the batch variable 1; *BV2* is the batch variable 2; and *ε*_*ijk*_ is the vector of residual errors. Wald tests were used to evaluate main effects while adjusting for the other factors and BV1/BV2. Differentially accessible regions (DAR) were considered statistically significant when they met the criteria of a *P*-value < 0.01 for the diet factor and a Benjamini-Hochberg (BH) adjusted *P*-value < 0.01 for the strain and age factors.

To assess diet effects within specific biological contexts (diet comparisons within a fixed strain and age), stratified analyses were performed by subsetting the dataset to each strain × age combination and refitting DESeq2 with:

$$y_{j}=\mu +\beta_{j}+BV1+BV2+\varepsilon_{j}\text{,}$$

where *y*_*j*_ is the response vector (for the specific strain × age subset); *μ* is the subset intercept; *β*_*j*_ is the effect of dietary P (fixed); *BV1* is the batch variable 1; *BV2* is the batch variable 2; and *ε*_*j*_ is the vector of residual errors.

Because strain and age are constant within each subset, they cannot confound the diet comparison; diet is therefore tested conditional on the given strain × age context . The batch variables BV1/BV2 were retained to control unwanted variation. Notably, stratification reduces sample size relative to the full factorial model and therefore reduces statistical power; subgroup analyses were treated as follow-up, context-specific contrasts. A nominal threshold of *P* < 0.01 was applied for these subgroup tests.

### 2.5 Differentially accessible peaks annotation

Venn diagrams were created for each applied contrast to display the DAR using the online tool jvenn (https://jvenn.toulouse.inra.fr/). Differentially accessible peaks were annotated to the nearest TSS using the HOMER annotatePeaks.pl (Heinz et al., 2010). Regions falling within 3 kb upstream to 200 bp downstream of the TSS were then classified as promoter regions, while all other regions were classified as distal regions. The nearest genes to DAR were used for functional analysis. Their enrichment in Gene Ontology Biological Processes (GO-BP) and Kyoto Encyclopaedia of Genes and Genomes (KEGG) pathways was examined, identifying key functional categories. Gene Ontology and KEGG pathway analyses were performed using the Database for Annotation, Visualization, and Integrated Discovery (DAVID) web-based functional annotation tool (https://davidbioinformatics.nih.gov/). Sequencing tracks were visualised using Integrative Genome Viewer (IGV) to examine the distribution of DAR and their relationships to genomic features. The enrichment of the signal peaks in the promoter regions of each experimental group was generated and visualised via the dba.plotProfile function in Diffbind (version 3.14.0).

### 2.6 Integrative analysis of gene expression and chromatin accessibility data

Previously published RNA-Seq data from the same samples were used for integrative analysis (Abitew et al., 2024). In brief, total RNA (approximately 50 mg jejunal mucosa; *n* = 80) was extracted with TRI Reagent (Sigma–Aldrich Chemie GmbH, Taufkirchen, Bavaria, Germany), DNase-treated, and QC'd by NanoDrop, Qubit, and Bioanalyzer (mean RIN 7.7 ± 0.6); Illumina mRNA-Seq libraries were prepared. Raw BCL files were converted to FASTQ (bcl2fastq v2.2.0), adapter-trimmed (Trim Galore v0.6.7), QC-checked (FastQC v0.11.9), and filtered (mean Q ≥ 20; length ≥ 30 bp) before alignment to GRCg6a with HISAT2 v2.2.0 (nf-core/RNA-Seq defaults; approximately 45.35 M reads/sample). Features were counted with htSeq-count v2.0.2 (union; Ensembl GTF), and batch effects were modelled with SVA/svaSeq v3.46.0 (BV1–BV2) alongside strain, diet, and age using DESeq2 package in the R environment (Love et al., 2014). Genes were differentially expressed at *P* < 0.01 for diet and BH-adjusted *P* (FDR) < 0.01 for strain and age. In total, 13,332 transcripts passed QC; differential expression was strongest by strain, followed by age and diet, with 44 transcripts significant across all factors and 3028, 3005, and 47 exclusive to strain, age, and diet, respectively.

Variance stabilising transformation (VST) was applied to both the ATAC-Seq and RNA-Seq datasets to normalise the data, reducing heteroscedasticity and enabling more accurate downstream analyses. Significant differentially accessible regions in the ATAC-Seq dataset, which were identified on the basis of the factors strain, diet, and age, and their corresponding differentially expressed genes (DEG) in the RNA-Seq dataset from the same samples were identified using the same threshold of a *P*-value < 0.01 for the diet factor and a Benjamini-Hochberg adjusted *P*-value < 0.01 for the strain and age factors. A Manhattan plot was generated to visualize significant DAR that corresponded with DEG across genomic regions. This visualization identified significant overlapping features between DAR and DEG, highlighting the most substantial coordinated changes in chromatin accessibility and gene expression across both assays.

To test whether overlap between DEGs and DARs exceeded chance expectations, over-representation analyses were performed using a one-sided Fisher's exact (hypergeometric) test implemented in clusterProfilerenricher (Bioconductor). The background (“gene universe”) was defined as the set of genes eligible for overlap detection-i.e., genes tested for differential expression in the RNA-Seq data and that could be linked to at least one ATAC-Seq peak under our peak-to-gene annotation strategy (*n* = 28,877 genes). *P*-values were adjusted for multiple testing using the Benjamini-Hochberg method within each contrast. Significance thresholds were: Diet contrasts, nominal *P* < 0.05; Strain and Age contrasts, FDR < 0.01 for both DEGs and DARs.

To infer the upstream regulatory drivers of the observed gene expression changes, ATAC-Seq derived regulatory potential was integrated with RNA-Seq expression data using ANANSE (v0.5.1) (Xu et al., 2021). The GRCg6a reference genome and gene annotation were used for all alignment and annotation steps. Condition-specific gene regulatory networks (GRNs) were constructed for each main effect. Transcription factor binding probabilities were predicted in ATAC-Seq peaks and combined with expression data to weight edges. By default, ANANSE uses the clustered vertebrate motif set gimme.vertebrate.v5.0, derived from CIS-BP (http://cisbp.ccbr.utoronto.ca/) and other sources, and benchmarked to perform well against ChIP-Seq peaks. To reduce noise, genes with near-zero expression (transcripts per million, TPM < 0.009) were filtered out prior to analysis. Finally, differential GRNs were computed by comparing the condition-specific networks. A TF influence score was calculated for each factor, quantifying how well the TF explains the differential expression of its target genes between conditions. The differential expression statistics used as input were the same as those described in the RNA-Seq analysis section.

### 2.7 Weighted gene Co-expression network analysis (WGCNA) and P- related phenotype

A large number of genes overlapping with ATAC peaks were identified, and WGCNA was further applied to examine whether these overlapping genes show stronger connectivity within co-expression modules in the P-related phenotypic traits. Gene co-expression networks were constructed using the WGCNA package (v.1.73) in the R environment (v. 4.4.2). The overlapping genes input matrix was normalized, and variance-stabilizing transformed counts were obtained. To achieve a scale-free network topology, the optimal soft-thresholding power (β) was determined using the ‘pickSoftThreshold’ function. Subsequently, gene modules were created using the 'blockwiseModules' function with the selected power (*β* = 12). The primary analysis parameters were set with a minModuleSize of 30, a deepSplit of 2, and a mergeCutHeight of 0.25. Finally, the module eigen (ME) was calculated, which represents the first principal component of each module's expression profile. The relationships between modules and traits were then quantified by correlating each ME with the measured phenotypic traits. These traits included body weight (BW), average daily feed intake (ADFI), P content in the jejunum, excreta, and plasma. The corresponding *P*-value determined the significance of each module–trait correlation. Additional transcripts from each module were submitted to analyze the biological processes (GO-BP) and pathways (KEGG).

## 3 Results

### 3.1 ATAC-Seq

**Fig. 1:**
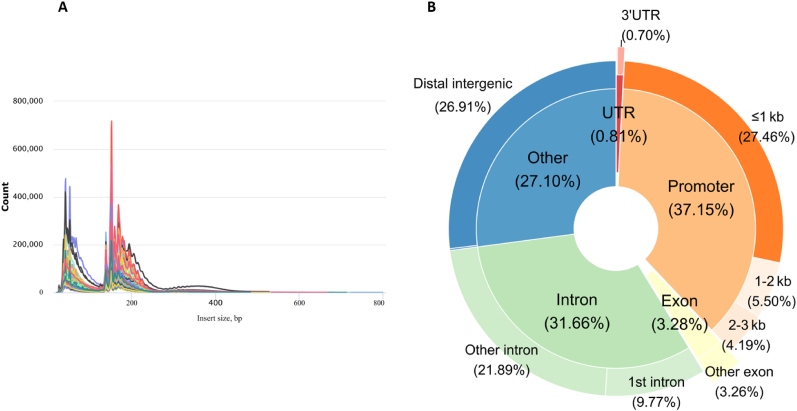
Assay for transposase-accessible chromatin using sequencing (ATAC-Seq) data quality and annotation. (A) Distribution of ATAC-Seq fragment (insert) lengths across 78 samples. (B) Comprehensive annotation of the gene regions of all peaks in the ATAC-Seq dataset from the jejunum mucosa of Lohmann Brown (LB) and Lohmann Selected Leghorn (LSL) hens in weeks 19 and 24. 3′UTR = three-prime untranslated region.

**Fig. 2:**
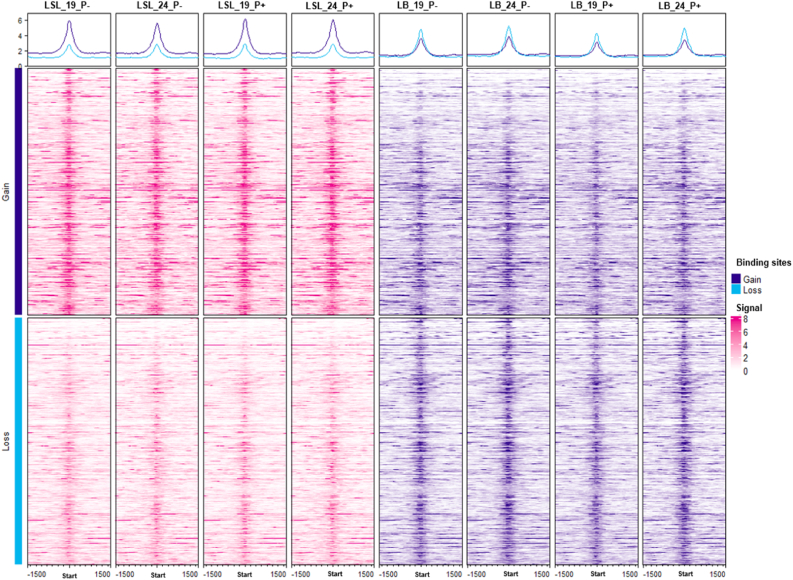
Heatmaps depicting assay for transposase-accessible chromatin using sequencing (ATAC-Seq) signal around all promoter regions, sorted by signal intensity. Peaks across regions were classified as having gained (top cluster) or lost (bottom cluster) accessibility between different experimental conditions. Each column corresponds to a specific time point and treatment/strain combination, with the top profile tracks illustrating the average signal over a ± 1.5 kb window around the promoter regions. The rows represent individual regions, enabling visualisation of how accessibility changes within each cluster compared across samples. Higher-intensity (darker) colours indicate a stronger ATAC-Seq signal. LB = Lohmann Brown; LSL = Lohmann Selected Leghorn; 19 and 24 denote the age (in weeks) at sampling, before and after the onset of egg-laying, respectively; P- = phosphorus (P)-deficient diet without mineral P supplement; P+ = P-supplemented diet (1 g/kg P from monocalcium phosphate).

The sequencing libraries were prepared from 80 jejunum mucosa samples, using a rigorous process that ensured high data quality. Two samples (LSL_week19_P+ and LB_week19_P-) had shallow sequencing coverage and were removed from further analysis. The alignment resulted in an average of 57.41 million fragment reads with a 97.94% mapping rate (Table S2). The fragment insert size distribution peaked at approximately 200 bp, with most fragments enriched in nucleosome-free regions (Fig. 1A). For the 78 samples, 2,727,995 peaks were identified (Table S2). Peak annotation revealed that 37.15% of the peaks were located in promoter regions (3 kb upstream and downstream of the TSS), followed by introns and other distal intergenic regions (Fig. 1B). A total of 286,062 consensus peaks were identified across all 78 samples. These peaks demonstrate strong consistency across all replicates, indicating the reproducibility of the data. Further profile plots were generated from these peaks using the diffBind R package with dba.plotProfile() to create complex heatmaps for each group, with a focus on promoter regions. Most losses and gains in chromatin accessibility within promoter regions were observed between strains rather than among age or diet groups (Fig. 2).

### 3.2 Differentially accessible regions between strains

**Fig. 3:**
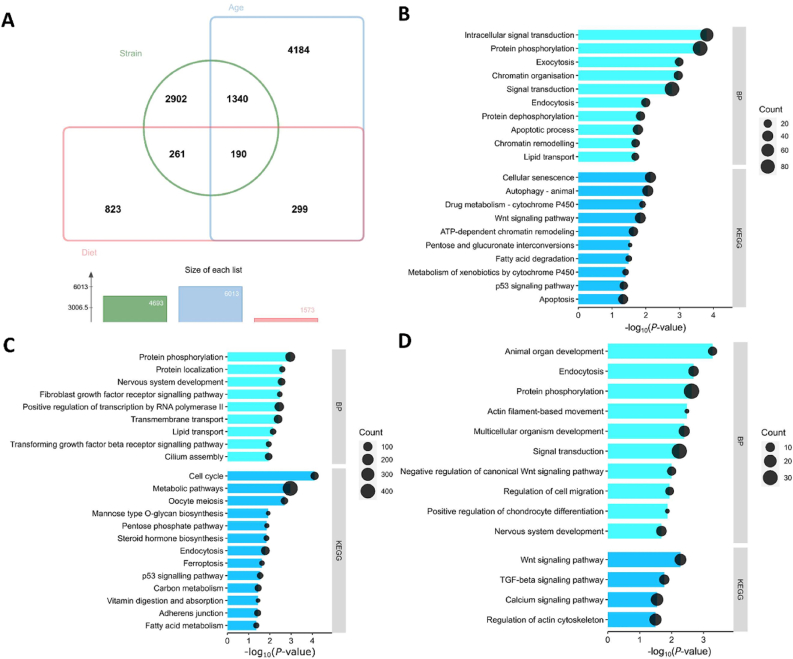
Effects of strain, age, and diet on chromatin accessibility. (A) Venn diagram displaying the overlap in differentially accessible regions (DAR) across strain (green), age (blue), and diet (red). The lower column plot shows the total number of DAR for each factor, comparing groups at an adjusted *P*-value < 0.01 for strain and age, and *P*-value < 0.01 for diet. (B) Bubble chart of Gene Ontology (GO) and Kyoto Encyclopedia of Genes and Genomes (KEGG) pathway analyses from DARs in the strain factor (LSL vs. LB); (C) in the age comparison (weeks 24 vs. 19), and (D) in the diet factor (P+ vs. P-). The x-axis represents the enrichment value [-log_10_ (*P*-value)]. The colour indicates enriched terms (light blue representing biological processes and dark blue KEGG pathways), and the size of the dots shows the number of enriched gene counts. LB = Lohmann Brown; LSL = Lohmann Selected Leghorn; P- = phosphorus (P)-deficient diet (without mineral P supplement); P+ = P-supplemented diet (1 g/kg P from monocalcium phosphate).

For downstream analysis, peaks with counts of 20 or more in at least five samples were retained, including 284,814 peaks corresponding to 28,056 nearest transcripts. Having established the sequencing quality and identified peaks, differential chromatin accessibility was examined across all the experimental groups. Pairwise comparisons of ATAC-Seq data using DESeq2 identified 6610 DAR corresponding to 4693 unique genes for the strain factor. The DAR were further categorized for each factor on the basis of positive (gain of accessibility) and negative (loss of accessibility) fold changes (Table S3). For the strain factor (LSL vs. LB), 3007 DAR were upregulated (gain of accessibility in LSL), and 3603 DAR were downregulated (loss of accessibility in LSL). Among the significant DAR in all the experimental factors (adjusted *P*-value < 0.01), 29% (2902 DAR) were unique to strain (Fig. 3A). Using HOMER's annotatePeaks.pl, genomic features were assigned to the significant DAR. For the strain factor, 7.59% of DAR were located in exons (502 DAR), 20.12% in intergenic regions (1330 DAR), 49.39% in introns (3265 DAR), 17.29% in promoter-TSS regions (1143 DAR), and 5.60% in transcription termination sites (TTS; 370 DAR) (Table S3).

The strain-based comparison revealed enriched pathways tied to intracellular signal transduction, protein phosphorylation/dephosphorylation, exocytosis/endocytosis, and chromatin organization/remodelling, with additional modulation of apoptotic processes, cellular senescence, autophagy, lipid transport, fatty acid degradation, and xenobiotic metabolism via cytochrome P450, alongside key roles for Wnt and p53 signalling pathways (Table S4 and Fig. 3B).

**Fig. 4:**
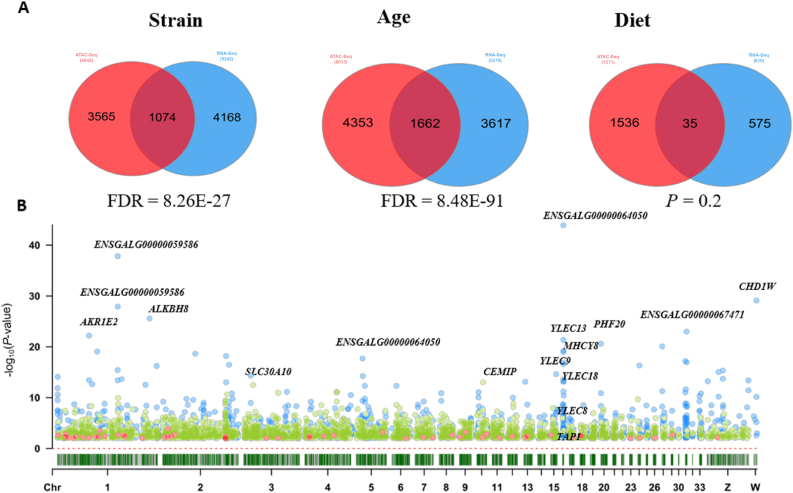
Integrative analysis of gene expression and chromatin accessibility data. (A) Venn diagrams (top) illustrate the overlap of significant differentially expressed genes (DEG) and differentially accessible regions (DAR) identified by RNA sequencing (RNA-Seq, blue circles) and assay for transposase-accessible chromatin using sequencing (ATAC-Seq, red circles) for each factor (strain, age, and diet) with their corresponding *P*-values. (B) The Manhattan plot (bottom) displays the -log_10_ (*P-*value) values for overlapping DAR in all three factors across chromosomes, with each factor represented by a distinct colour (Strain = blue, Age = green, Diet = red). Labelled genes indicate notable hits based on the ATAC-Seq data (*P* < 0.01).

**Fig. 5:**
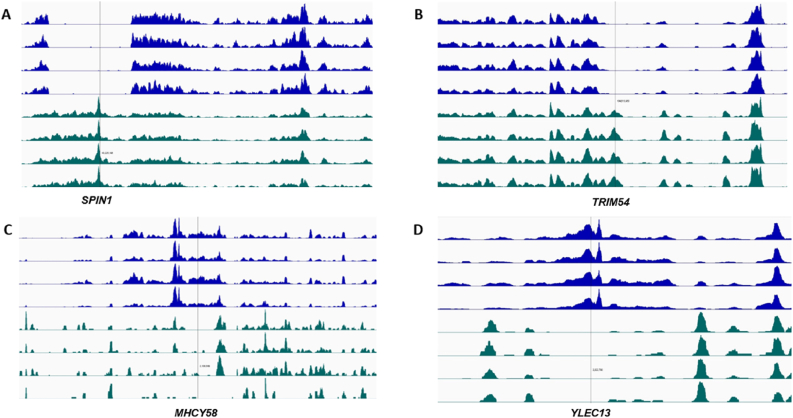
Visualisation of differential accessible regions (DAR) between hen strain of (A) *SPIN1*: involved in chromatin regulation and transcriptional control, (B) *TRIM54*: associated with protein ubiquitination and muscle function, (C) *MHCY58*: involved in immune signalling and antigen presentation, and (D) *YLEC13*: a C-type lectin-like gene that may mediate immune recognition and response. Blue track representing Lohmann Brown (LB) hens and the dark green track representing peaks from Lohmann Selected Leghorn (LSL) hens.

Further, previously published RNA-Seq data was integrated (Abitew et al., 2024) with ATAC-Seq data from the same experimental animals. The downstream analysis focused exclusively on the statistically significant results that overlapped between the two datasets and ensured that each overlapping result showed the exact directional change. A total of 1074 overlapping transcripts associated with the strain factor were identified (Fig. 4A), 226 of which had ATAC-Seq peaks in their promoter regions (Table S5). Among the genes with significant changes in chromatin accessibility, distinct patterns were observed between the LSL and LB strains. In LB hens, increased accessibility in the promoter regions was observed for the C-type lectin-like protein family members *YLEC8*, *YLEC9*, and *YLEC13*, as well as for *TAP1* (The full names of the abbreviations of all genes can be found in supplementary file abbreviations and full names of genes). Although *YLEC18* is part of the same gene family, its increased accessibility was detected in an intergenic region (Fig. 4B). Increased promoter accessibility for *CHIR3B10*, *SAAL1*, *REX05*, *ETV7*, *TNFRSF18*, *UNC13D*, *HCK*, *DENND1B*, *CD274*, and *RUNX3* was observed in LSL hens. *SPIN1*, *TRIM54*, *PHAX*, and *RECQL4* exhibited markedly greater chromatin accessibility in LSL compared to LB, whereas *YLEC13*, *AKR1E2*, *YLEC9*, and *MHCY58* presented a more pronounced DAR opening in LB compared to LSL. Among these, the four genes *SPIN1*, *TRIM54*, *YLEC13*, and *MHCY58* are shown in IGV as representative examples (Fig. 5A–D). For readability, Fig. 4B labels only a curated subset of the most prominent significant hits; the remaining significant genes discussed here are reported in Table S5.

### 3.3 Differentially accessible regions between production periods

Analyses revealed about 7884 DAR (6013 unique genes) for the age factor (weeks 24 vs. 19), where 3687 DAR were upregulated (increase in accessibility in the week 24), and 4193 DAR were downregulated (loss of accessibility in the week 24). Among all significant DAR in all of the experimental factors, 41.8% (4184 DAR) were unique to age (Fig. 3A). Genomic features were assigned to the significant DAR of the age factor, where 8.54% of DAR were in exons (673 DAR), 13.12% were in intergenic regions (1034 DAR), 43.62% were in introns (3439 DAR), 29.36% were in promoter-TSS regions (2315 DAR), and 5.37% were in TTS (423 DAR) (Table S3).

The age-based comparison (weeks 24 vs. 19) revealed enriched pathways associated with protein phosphorylation, localization, nervous system development, and growth factor receptor signalling, alongside shifts in transcriptional regulation, transmembrane and lipid transport, and cell cycle control. The DAR during the maturation period were prominently observed in metabolic pathways. Key metabolic routes, including carbon metabolism, the pentose phosphate pathway, fatty acid metabolism, and steroid hormone biosynthesis were significantly altered throughout the transition from weeks 19 to 24 (Table S4 and Fig. 3C).

A total of 1662 overlapping transcripts related to the age factor were identified when compared with previously published RNA-Seq data (Fig. 4A), with 415 showing peaks in the promoter regions (Table S6). Increased promoter accessibility was observed at week 19 (345 transcripts) for a wide range of genes belonging to the cell cycle process, including *CCNB1*, *CCNE2*, *CDC20*, *CDC7*, *CDK1*, *CUL1*, *DBF4*, *DDX11*, *MCM5*, *MCM6*, *MYC*, *ORC6*, *PLK1*, *PTTG2*, *SGO1*, and *TGFB3*. Other increased promoter accessibility of week 19 was enriched in phosphatidylinositol signalling system including *DGKZ*, *INPP1*, *ITPR1*, *MTMR1*, *PIK3CD*, *PIP4K2A*, *PLCG1*, and *PRKCB*. The top 10 that increased promoter accessibility at week 19 were *GPC1*, *NRP1*, *ADAMTS14*, *PKDCC*, *LPCAT2*, *SYK*, *MOXD1*, *DCLK2*, *TSPAN4*, and *PALD1*. Only 70 DAR showed an overlap with RNA-Seq data and were located in promoter regions. An enrichment was observed in metabolic pathways (*ADA*, *AKR1D1*, *CA3B*, *ENPP4*, and *ENPP8*), *ENTPD8L1*, *FUT9*, *GUCY2C*, *HKDC1*, *MDH1*, and *MGAT4C*. Most increased promoter accessibility at week 24 included *CA3B*, *EI24*, *UPK1B*, *EDA*, *CALB1*, *FGFBP1*, *SKP1*, *TASOR2*, *HKDC1*, and *RAPGEFL1*.

### 3.4 Differentially accessible regions between diets

About 1671 DAR (1573 unique genes) for the diet factor were observed. For the diet factor (P+ vs. P-), 541 DAR were upregulated (gain of accessibility in the P+ group), and 1130 DAR were downregulated (loss of accessibility in the P+ group). Among all significant DAR in all the experimental factors, 8.2% (823 DAR) were unique to diet (Fig. 3A). For genomic features of the diet factor, 8.02% of DAR were in exons (134 DAR), 22.3% in intergenic regions (373 DAR), 55.83% in introns (933 DAR), 8.80% in promoter-TSS regions (147 DAR), and 5.03% in TTS (84 DAR) (Table S3).

Differentially accessible regions in the diet-based comparison (P+ vs. P-) showed enriched pathways linked to endocytosis, protein phosphorylation, multicellular organism development, signal transduction, and nervous system development. Analysis of the Kyoto Encyclopedia of Genes and Genomes (KEGG) further identified enrichment in the Wnt, transforming growth factor-β (TGF-β), calcium signalling pathways, and the regulation of the actin cytoskeleton (Table S4 and Fig. 3D). Regarding the diet factor, the analysis revealed 35 overlapping transcripts between RNA-Seq data and ATAC Seq data (Fig. 4A), four of which had peaks in promoter regions (Table S7). Specifically, *SH3BGRL*, *PMP22*, *ENSGALG00000048239*, and *CALB1* were observed to have significantly different chromatin accessibility in their promoter regions. The Manhattan plot displays the –log_10_ (*P*-values) for overlapping DAR in all three factors across chromosomes, with each factor represented by a distinct colour (Fig. 4B).

### 3.5 Differentially accessible regions between diets within strain and production periods

**Fig. 6:**
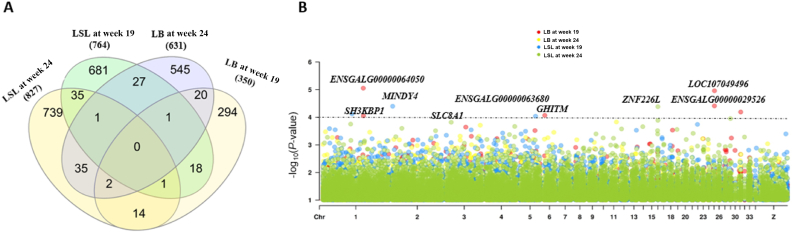
Effects of diets on chromatin accessibility in laying hen strains at different production periods. (A) Venn diagram showing differentially accessible regions (DAR) between diets lacking mineral phosphorus (P) supplement (P-) and those with P supplement (P+) across four hen strain and production period groups: Lohmann Selected Leghorn (LSL) at week 24 , LSL at week 19 , Lohmann Brown (LB) at week 24, and LB at week 19. (B) Manhattan plot displaying –log_10_ (*P*-value) values for overlapping DAR across chromosomes, with each group represented by a distinct colour: LSL at week 24 = green, LSL at week 19 = blue, LB at week 24 = yellow, and LB at week 19 = red. DAR with -log_10_ (*P-*value) values greater than 4 are labelled.

**Fig. 7:**
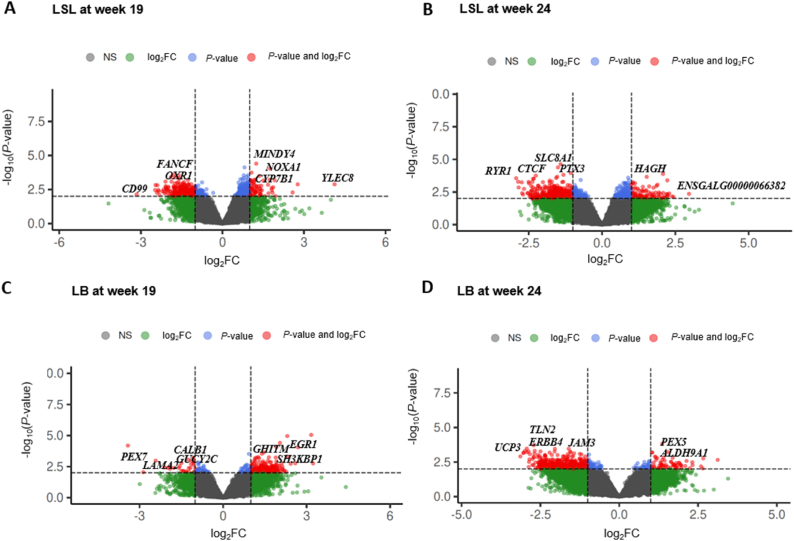
Volcano plots of differentially accessible regions (DAR) between diets lacking mineral phosphorus (P) supplement (P-) and those with P supplement (P+) across four hen strain and production period groups: (A) Lohmann Selected Leghorn (LSL) at week 19, (B) LSL at week 24, (C) Lohmann Brown (LB) at week 19, and (D) LB at week 24. Significant DAR (*P* < 0.01) with a |log_2_ FC| > 1 are highlighted in red. FC = fold change; NS = not significant.

At week 19, LB hens exhibited 301 DAR with higher accessibility in the P+ group, compared to 71 DAR with higher accessibility in the P- group (Fig. 6A and Table S8). Similarly, in LSL hens at the same age, 315 peaks were more accessible in the P+ group, whereas 487 DAR showed higher accessibility in the P- group (Fig. 6A and Table S8). By week 24, greater accessibility was generally observed in the P- diet group. In LB hens, 660 DAR were identified (Fig. 6A), with 94 showing higher accessibility in the P+ group and 566 in the P- group (Table S8). In LSL hens, 867 DAR were significantly different between the dietary groups (Fig. 6A), with 276 exhibiting higher accessibility in the P+ group and 591 in the P- group (Table S8). In total, 2687 DAR that were significant in at least one group were visualized in the Manhattan plot, displaying –log_10_ (*P*-value) values for overlapping DAR across chromosomes (Fig. 6B). Several significant DAR were identified in the LSL hens at week 19 (Fig. 7A), which belongs to gut structure and metabolism. These include *TIMP3*, *CYP7B1*, *NOXA1,* and *TPH2*. In LSL hens at week , the DAR mapped predominantly to genes modulating calcium handling, intracellular trafficking and signalling. These significant DAR include *SLC8A1*, *RYR1*, *EPHB2*, *DUSP6,* and *UCP3* (Fig. 7B). LB hens at week 19 showed significant DAR associated with ion transport, epithelial barrier components and nutrient processing. Among of these significant DAR were *SLC9A2*, *CALB1*, *GUCY2C*, *LAMA2*, and *DGAT2* (Fig. 7C). The DAR profile in LB hens at week 24 pointed to genes involved in epithelial integrity, signalling pathways and energy metabolism. Among the DAR identified, *JAM3*, *TLN2*, *ERBB4*, *DUSP6,* and *UCP3* were found to be significantly altered (Fig. 7D).

### 3.6 Transcription factor influence signals and gene regulatory networks (GRNs)

**Fig. 8:**
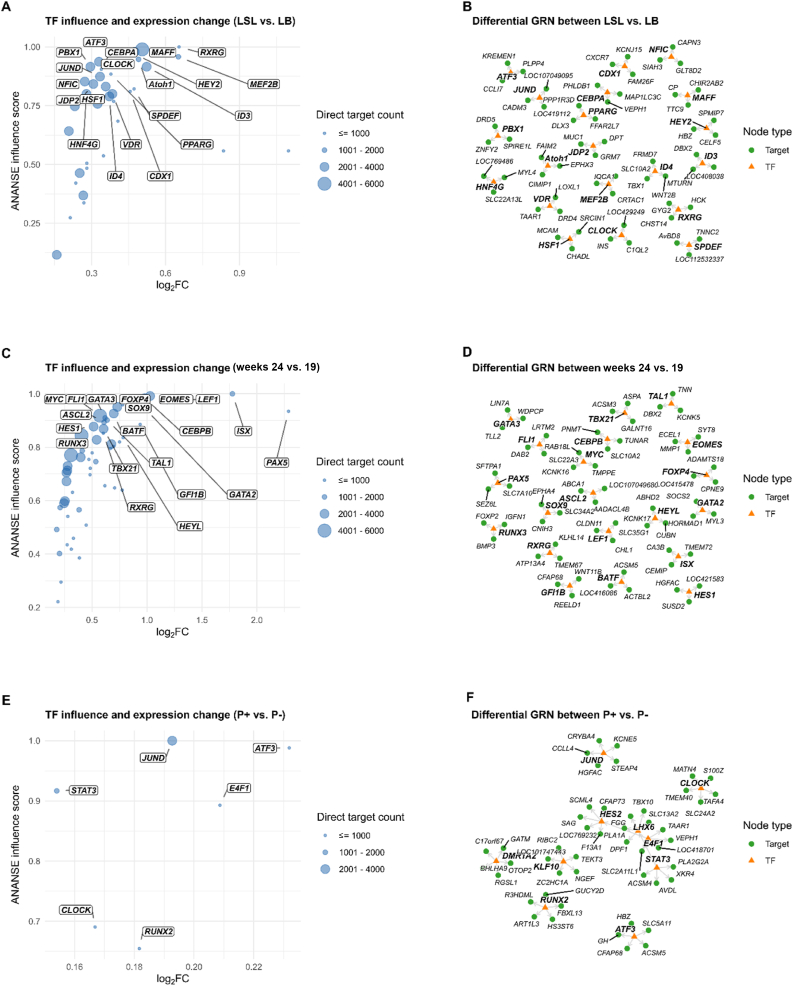
Transcription factor (TF) influence and differential gene regulatory networks (GRNs) across strain, age, and diet contrasts. (A, C, and E) Scatterplots summarising ANalysis Algorithm for Networks Specified by Enhancers (ANANSE) TF influence scores (y-axis) against the corresponding RNA sequencing (RNA-Seq) log_2_FC of each TF (x-axis) for the indicated comparisons: LSL vs. LB (A), weeks 24 vs. 19 (C), and P+ vs. P- (E). The top influential TFs are labelled, and the bubble size represents the number of direct target genes for that TF in each contrast. (B, D, and F) Differential GRNs inferred by ANANSE for the same contrasts shown in (A, C, and E), respectively. Networks display predicted TF-target regulatory relationships, with nodes coloured by class (TFs vs. target genes; see legend). Edges represent differential regulatory interactions between conditions in each comparison, highlighting dominant TF hubs and rewired target connections. LB = Lohmann Brown; LSL = Lohmann Selected Leghorn; FC = fold change; weeks 24 vs. 219 denote the age at sampling; P- = phosphorus (P)-deficient diet (without mineral P supplement); P+ = P-supplemented diet (1 g/kg P from monocalcium phosphate).

Consistent with the transcriptomic and chromatin accessibility contrasts, strain and age effects produced clear TF influence signals and structured differential GRNs, whereas the diet effect was notably more modest. The comparison between strains (LSL vs. LB) highlighted a set of high-influence TFs driving strain-specific expression differences. Prominent drivers included *RXRG*, *CEBPA*, *MAFF*, *PBX1*, *ATF3*, and *JUND* (Fig. 8A). The GRN identified *RXRG* as a top regulatory hub distinguishing the LSL and LB strains (Fig. 8B). Complementing the metabolic regulation by *RXRG*, *RXRG* was also observed to target *GYG2*, an enzyme for glycogen formation. In the network analysis, *RXRG* was connected to genes such as *HCK*, a kinase associated with hematopoietic and innate immune pathways, and *CHST14*, a carbohydrate sulfotransferase influencing proteoglycan structure, suggesting potential cross-talk between *RXRG* signalling, immune function, and extracellular matrix remodelling. The GRN highlighted *PPARG* as a key driver connected to *PPP1R3D*, a regulatory subunit of protein phosphatase 1 involved in carbohydrate/glycogen metabolism.

Significant regulatory shifts associated with maturation were identified (weeks 24 vs. 19). Among the top TFs influencing this transition were developmental and differentiation factors, including *GATA3, EOMES, PAX5, ISX, GATA2, LEF1*, and *FOXP4* (Fig. 8C). The GRN highlights the specific targets of these developmental hubs (Fig. 8D). Network topology analysis further indicated that *EOMES* targets *MMP1*, a matrix-associated protein involved in tissue organisation and remodelling. The regulatory factor *ISX* was predicted to target *CEMIP* (*KIAA1199*), and *FOXP4* emerged as a key regulator targeting *ADAMTS18*, a metalloproteinase implicated in extracellular matrix organisation.

In contrast to the extensive network topology observed in strain and age, the diet contrast (P+ vs. P-) yielded a sparse, specific regulatory network. The top ranked transcription factors included the osteogenic master regulator *RUNX2* and stress responsive factors such as *ATF3*, *JUND*, and *STAT3* (Fig. 8E). The GRN showed specific regulatory edges linking these factors to solute transport and metabolic buffering (Fig. 8F). Specifically, *RUNX2* was found to target *HS3ST,* and *GUCY2D*. A direct link between circadian factor *CLOCK* and *SLC24A2* was also identified. Furthermore, *LHX6* targeted *SLC13A2* while *STAT3* targeted *PLA2G2A*.

### 3.7 Integrating overlapping DEGs and DARs with P-related phenotypes

Considering phenotypic traits, body weight, ADFI, and P concentrations in jejunal digesta, excreta, and plasma (Table 1), LB hens were significantly heavier than LSL hens (*P* < 0.001), and hens at week 24 were heavier than those at week 19 (*P* < 0.001). P concentrations in jejunal digesta and excreta did not differ significantly between strains (*P* > 0.05), whereas plasma P was higher in LB than in LSL (*P* = 0.010). Dietary P level had no significant effect on body weight, ADFI, or P in excreta (*P* > 0.05); however, P+ increased P concentrations in both jejunal digesta and plasma relative to P- (*P* < 0.05).

**Table 1:** Effects of hen strain, production period and dietary phosphorus (P) level on BW, ADFI, concentration of P in jejunum content, excreta, and plasma.

Item	Strains^1^		Age (week)		Diets^2^		SEM	*P-*value			
	LB	LSL	19	24	P+	P-		Strain	Age	Diet	Strain × age × diet
BW^3^, g	1612.6	1298.6	1257.8	1653.5	1463.7	1447.6	22.78	<0.001	<0.001	0.484	0.349
ADFI^4^, g/d	81.9	78.1	83.0	77.0	80.1	79.9	1.92	0.071	<0.001	0.460	0.369
Jejunum-P, g/kg DM	9.1	8.7	9.8	8.0	9.7	8.2	0.32	0.119	<0.001	<0.001	<0.001
Excreta-P, g/kg DM	11.2	10.6	9.4	24.4	11.3	10.5	0.55	0.250	<0.001	0.071	0.529
Plasma-P, mg/dL	5.4	4.8	6.1	4.1	5.8	4.3	0.22	0.010	<0.001	<0.001	0.072

BW = body weight; ADFI = average daily feed intake.

Differences were considered significant at *P* < 0.05. Data are given as means and standard error of the mean (SEM), *n* = 10 hens.

1 LB, Lohmann Brown; LSL, Lohmann Selected Leghorn.

2 P+, P-supplemented diet (1 g/kg P from monocalcium phosphate); P-, P-deficient diet (without mineral P supplement).

3 BW until the day before slaughter.

4 ADFI in the whole period (4 weeks) until the respective day of slaughter.

**Fig. 9:**
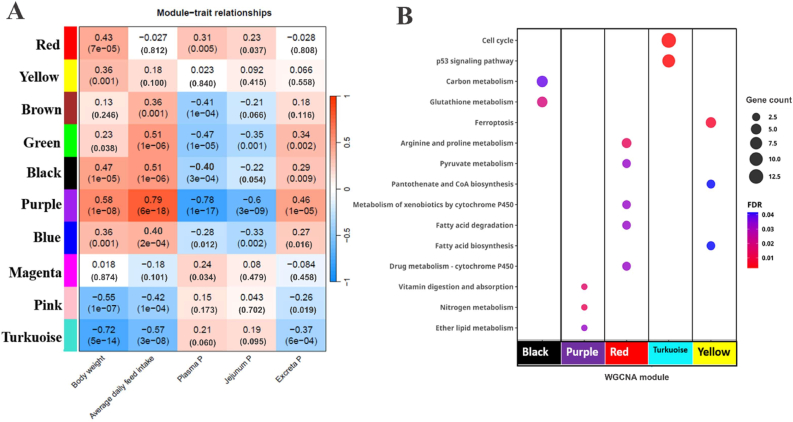
Weighted gene co-expression analysis (WGCNA). (A) Module–trait relationships. Heatmap shows Pearson correlations (*r*) between module eigengenes and phenotypes (body weight, average daily feed intake, plasma phosphorus level, jejunum phosphorus level, and excreta phosphorus level); each cell is *r* (*P*-value). Red/blue indicate positive/negative associations, and the side colour bar denotes the WGCNA module (red, yellow, brown, green, black, purple, blue, magenta, pink, and turquoise). (B) Module functional analysis: Kyoto Encyclopedia of Genes and Genomes (KEGG). Dot plot of enriched KEGG pathways for the indicated modules. Dot colour = false discovery rate (FDR; Benjamini-Hochberg adjusted *P*-value), dot size = Gene Count (number of module genes in the pathway); vertical guide lines separate modules. Only pathways with FDR < 0.05 are displayed.

Phenotypic traits were integrated with the transcriptomic dataset by prioritizing features supported by concordant evidence from both differential expression and chromatin accessibility (i.e., genes overlapping between DEGs and DAR-linked genes). The overlap between DEG and DAR was highly significant for strain and age (FDR < 0.001), exceeding chance expectations. Diet did not reach the significance threshold; however, genes linked to increased accessibility (DAR_UP) showed nominal enrichment, whereas genes linked to decreased accessibility (DAR_DOWN) showed weaker enrichment. In total, 1778 transcripts met the overlap criteria and used for WGCNA. The analysis identified 10 co-expression modules (Fig. 9A). Two modules, purple and turquoise, emerged as key players due to their pattern with growth and P related-traits. The purple module with 51 genes showed significant positive correlations with both ADFI (*r* = 0.79, *P* < 0.001), body weight (*r* = 0.58, *P* < 0.001), and P content in excreta (*r* = 0.46, *P* < 0.001). Conversely, it was negatively correlated with plasma P (*r* = −0.78, *P* < 0.001) and P content jejunum digesta (*r* = −0.6, *P* < 0.001). In contrast, the turquoise module with 378 genes was negative correlated with body weight (*r* = −0.72, *P* < 0.001), ADFI (*r* = −0.57, *P* < 0.001) and P content in excreta (*r* = −0.37, *P* < 0.001), while showing a tendency toward positive correlations with P content in jejunal digesta and plasma. Genes in the purple module were significantly enriched for pathways related to nitrogen metabolism, lipid metabolism, and vitamin digestion and absorption, whereas the turquoise module was enriched for cell cycle and p53 signalling pathways (Table S9 and Fig. 9B). The black module with 141 genes showed a correlation pattern similar to the purple module and was enriched for carbon and glutathione metabolism. Genes in other modules with the same correlation pattern (brown, green, and blue) did not reach significance in enrichment analysis at FDR < 5%. The red and yellow modules, containing 146 and 181 genes, respectively, showed significant positive correlations with body weight; notably, the red module was additionally positively correlated with P content in plasma and jejunal digesta. Genes in both modules were enriched for metabolic categories, including amino acid, lipid, carbohydrate, and energy metabolism (Table S9 and Fig. 9B).

## 4 Discussion

Epigenetic regulation and particularly chromatin accessibility studies related to strain and the maturation period of poultry have provided insight into gene expression regulation (Dunislawska et al., 2022). While studies on effects of variable dietary P supply were missing, the genetic effects on P utilisation and related traits have been comprehensively reported, revealing significant differences between LSL and LB laying hen strains (Reyer et al., 2021; Sommerfeld et al., 2020). Age-related effects on gene expression have also been noted, particularly in relation to the onset of egg laying (Abitew et al., 2024; Reyer et al., 2021). Based on previous findings of strain-specific jejunal gene expression (Abitew et al., 2024) and phenotypic as well as physiological changes in response to dietary P levels (Qasir et al., 2025; Sommerfeld et al., 2024), this study investigated chromatin accessibility as an additional layer of epigenetic regulation. Significant strain effects were observed, with over 6000 genomic regions showing notable differences in chromatin accessibility between the two strains. The LSL genome exhibited less chromatin accessibility (3603 DAR) compared to the LB strain. A strain effect in the same cohort was also evident at both the physiological and transcriptomic levels (Abitew et al., 2024;; Sommerfeld et al., 2024;). Together, these findings suggest that, beyond genetic differences, epigenetic regulation also contributes to the distinct phenotypes of these strains. Similar chromatin accessibility differences between breeds have also been observed in various species, providing further insights into genetic regulation and trait variation (Lee and Cho, 2025).

The strain-specific chromatin remodelling indicates the inherent genetic influences on transcriptional regulation, incl. immune function (Dahmer et al., 2016; Morrison, 2020). In fact, immune-related genes (*MHCY8*, *MHCY58*, *YLEC9*, *YLEC19*, and *YLEC18*) suggested that strain differences may impact disease resistance and immune responsiveness (Goto et al., 2022). In addition, genes involved in phospholipid signalling (*PLCB4*) (Rebecchi and Pentyala, 2000) and glucose metabolism (*PGM2L1*) (Morava et al., 2021) showed variations that may affect nutrient use efficiency, while *SEC14* is involved in intracellular trafficking (Curwin et al., 2009). Higher chromatin accessibility in the promoter regions and increased expression of the C-type lectin-like protein family in LB, including *YLEC8*, *YLEC9*, and *YLEC13* were observed. This finding is in line with a recent study on the differential expression of C-type lectin-like proteins in LB and LSL hens, which suggests that LB chickens may have enhanced immune competence and disease resistance, as indicated by lower virus shedding and distinct immune responses to *H9N2* avian influenza infection (Youk et al., 2024). In addition, *TAP1*, which plays a crucial role in antigen processing and presentation via MHC class I, also exhibited higher accessibility of the corresponding promoter region and increased expression in LB compared to LSL. The MCH-Y family contains genes involved in innate and adaptive immunity in chickens (Kaufman, 2022). The pronounced significant DAR at *MHCY58* in LB compared to LSL suggests a potential role in regulatory or immune-related functions unique to that strain (Yuan et al., 2021). On the other hand, increased promoter accessibility and higher expression of *ETV7*, *TNFRSF18*, *UNC13D*, *HCK*, *DENND1B*, *CD274*, and *RUNX3* were observed in LSL hens compared to LB hens. Most of these genes are involved in immune responses. Specifically, *TNFRSF18*, *DENND1B*, *CD274*, and *RUNX3* are associated with the activation and regulation of T cells (Beenen et al., 2022; Cruz-Guilloty et al., 2009; Tian et al., 2020; Yang et al., 2016); *ETV7* is involved in interferon signalling (Froggatt et al., 2021); *HCK* is associated with myeloid cell activation (Carvalho et al., 2024); and UNC13D mediates the exocytosis of cytotoxic granules in natural killer (NK) cells and cytotoxic T cells (Krzewski and Coligan, 2012). Hematopoietic cell kinase, a member of the Src family of *NRTK*, is involved in various inflammatory responses (English et al., 1993). In this previous study, higher *HCK* expression was found in the LSL strain compared to LB, with a positive correlation to total T-cell and CD8-γδ-T cell counts in laying hens (Ponsuksili et al., 2023). This study again confirms that the innate and humoral immune response is more pronounced in LB hens, while LSL's genetic profile favours adaptive cellular defences (Hofmann et al., 2021; Schmucker et al., 2021). Functionally, these patterns suggest that LB maintains a more “innate-forward” epithelial state that may favour rapid luminal sensing and transport adjustments, while LSL emphasizes adaptive cellular regulation, two distinct immune settings that can secondarily shape transporter expression and barrier remodelling in the gut. Integrative GRN analysis of ATAC-Seq and RNA-Seq (ANANSE) indicates that LB-LSL differences involve upstream regulatory rewiring as well as downstream expression shifts. The network prioritised *RXRG* and predicted *RXRG* regulation of immune signalling genes including *HCK* (English et al., 1993), consistent with evidence that *RXRγ* restrains small-intestinal *ILC2* responses by maintaining cholesterol efflux programs (Zang et al., 2023). In addition, *RXRG*–*PPARG* emerged as central drivers, converging on glycogen machinery (*GYG2* and *PPP1R3*) and implicating altered energy handling (Plutzky and Kelly, 2011; Royan and Navidshad, 2016). Signalling through *PPARG* support epithelial metabolic and barrier regulation (Hontecillas and Bassaganya-Riera, 2012; Noori et al., 2024), while *CEBPA* enrichment suggests contributions from innate immune programs and possible breed differences in immune-cell activation/composition (Lee et al., 2014; Liu and Lee, 2013).

Notably, 13.4% of DAR (1340 DAR) overlapped between strain and age effects, indicating that strain-specific chromatin accessibility changes moderately reflect differences in developmental timing. This overlap suggests that the observed chromatin remodelling patterns are not exclusively driven by genetic background but are also influenced by age.

The analyses of chromatin accessibility profiles in laying hens transitioning from weeks 19 to 24 revealed over 6000 DAR, highlighting extensive epigenetic remodelling during this developmental shift. Across age, enrichment of cell-cycle/growth-factor, transmembrane and lipid transport, and central metabolic pathways (carbon metabolism, pentose phosphate, fatty-acid metabolism, and steroid biosynthesis) indicates a global rewiring that accompanies the transition to egg-laying. Differential Accessible Regions overlapping with DEG at *CALB1, SLC25A24, FAM20A, DGAT2, INHBB* and others point to a shift from a more proliferative program at week 19 (enhanced cell-cycle/promoter accessibility) toward a transport-competent, energy-intensive epithelial phenotype by 24 weeks, supporting increased mineral flux (Ca/P) and steroid/lipid handling during onset of lay.

Increased accessibility at these loci suggests that epigenetic mechanisms may be crucial in phosphate and calcium utilisation, with implications for nutrient absorption, bone mineralisation, and eggshell formation (Zhang et al., 2017). Consistent with previous published results on maturation effects, the demand for P and calcium increases as hens’ approach peak egg production, which is associated with changes in intestinal gene expression and structural adaptations (Shi et al., 2024; Sinclair-Black et al., 2023). Concurrently, the immune system undergoes significant shifts between weeks 15/16 and 23/24, coinciding with the transition to egg-laying activity (Schmucker et al., 2021). Integration of DAR and RNA-Seq data revealed genes enriched in pathways related to regulation, development, homeostasis, and phosphorus metabolism, reflecting metabolic shifts at the onset of egg-laying (Garcia-Mejia et al., 2024; Sinclair-Black et al., 2023).

Analysis of age-dependent effects (weeks 19 vs. 24) clearly shows how chromatin remodelling shapes biological function. Developmental transcription factors such as *GATA3*, *ISX*, and *EOMES* emerged as dominant regulators. Given that *GATA3* and *EOMES* are key drivers of mucosal innate and adaptive lymphocyte programs that maintain gut barrier homeostasis (Lin et al., 2023; Tindemans et al., 2014), and *ISX* participates in intestinal epithelial differentiation (Lobo et al., 2010), their prominence is consistent with mucosal remodelling during transition to lay potentially reflecting coordinated epithelial and immune reorganisation (Ponsuksili et al., 2023). This rewiring likely involves structural remodelling and metabolic specialization (Shi et al., 2024). The transcription factor *ISX,* a key regulator integrating diet-mediated signalling with intestinal programs (Widjaja-Adhi et al., 2017), was predicted to target the matrix regulator *CEMIP* (Liu et al., 2023), consistent with jejunal molecular transitions at lay onset in high-yield layers (Omotoso et al., 2021). The regulator *FOXP4* recruits *ADAMTS18*, a gene involved in remodelling the extracellular matrix (Nie and Zhang, 2023). This kind of regulation can affect how the intestinal tissue is structured and maintained, helping shape tissue integrity during the maturation period (Hrabia et al., 2022; Omotoso et al., 2021). The strong influence of these factors suggests that differential gene expression is a downstream effect of this upstream remodelling (Voss and Hager, 2014).

A previous study showed that most DEGs were driven by strain and age, with minimal effects from dietary P (Abitew et al., 2024). Similarly, changes in chromatin accessibility due to P supplementation were subtle, suggesting no adverse impact on the jejunal mucosa within this maturation window. However, KEGG analysis of diet-based DARs showed enrichment of Wnt, TGF-β, and calcium signalling pathways. In line with coordinated reprogramming under mineral restriction, accessibility was reduced at several pro-proliferative, Wnt-related genes in the P- group (*MAPK10, VANGL1, DKK4,* and *PPP3CA*), consistent with diminished intestinal stem-cell renewal. By contrast, loci linked to cell-cycle restraint and TGF-β signalling (e.g., *TGFB1, CDKN1B,* and *SMAD*-family genes), as well as negative regulators of Wnt (*GSK3A/B*), were more open, suggesting engagement of anti-proliferative programmes (Huang et al., 2022; Oost et al., 2022). Among the diet-responsive genes, those involved in calcium metabolism (*CALB1* and *RGN*) and phosphate metabolism (*FGGY* and *PAPSS*) were the most prominent, which aligns with their direct roles in mineral homeostasis and eggshell formation. Genes linked to cell growth and stress responses (*JUND*) and broader metabolic/signalling pathways (*GSK3A*, *SLC26A2*, and *PREP*) suggest that certain transcriptional regulators remain responsive to mineral P removal. The transcription factor *JUND* is induced by metabolic stressors such as polyamine depletion in intestinal epithelial cells and regulates genes involved in epithelial proliferation and barrier function, indicating a role in epithelial stress responses in the gut (Chen et al., 2008; Xiao et al., 2007). The expression of genes involved in bone/cartilage remodelling and phosphorylation/signalling regulation may help laying hens to adapt to fluctuating dietary mineral availability. Specifically, *ADAMTS7*, *RGN*, *CALB1*, and *SLC26A2* support ECM maintenance and regulate calcium/P homeostasis, potentially safeguarding skeletal integrity under challenging mineral P supplementation (Sun et al., 2020). Among the significant DAR, chromatin accessibility was significantly increased for *CALB1* and *DECR1* under the P- diet, whereas *UTS2R*, *GHITM*, *KLHL4*, and *SLC26A2* exhibited higher accessibility in P+. These distinct patterns suggest that dietary P levels trigger specific regulatory changes that may impact intestinal mineral absorption (Sinclair-Black et al., 2023). The ANANSE algorithm integrates predicted TF binding in accessible chromatin with expression data to weight GRN edges and rank TFs by influence (P+ vs. P-), highlighting and consistent with regulators linked to bone/cartilage remodelling (*RUNX2*, *STAT3*, and *CLOCK*) (Liu and Lee, 2013; Yuan et al., 2017; Zhou et al., 2021) and stress/AP-1 signalling (*ATF3* and *JUND*) (Chen et al., 2008; Hai et al., 1999; Xiao et al., 2007).

Despite the subtle effects of dietary P, chromatin changes were examined to identify strain- and age-specific responses. The ATAC-Seq analysis revealed distinct, context-dependent epigenetic modulation in the jejunum. In LB hens at week 19, chromatin accessibility at *CALB1, GUCY2C*, and *GRAMD1C* (Aster-C) was higher under the P-deficient diet (P-). This aligns with our previous finding that dietary P strongly affected plasma inorganic phosphorus, particularly at week 19 under P- (Qasir et al., 2025), indicating measurable physiological responses and strain differences in P utilisation. Functionally, *GRAMD1C* supports cholesterol transport from the plasma membrane to the ER in enterocytes and influences dietary cholesterol absorption (Sandhu et al., 2018). The *CALB1* gene encodes calbindin-D28K, facilitates intracellular calcium transport across intestinal epithelial cells, and its expression is regulated by both diet and environmental factors (Laptev et al., 2021; Theofan et al., 1987). The receptor *GUCY2C* regulates fluid and electrolyte balance by modulating ion transport through cGMP-dependent signalling, which is essential for proper gut function (Lan et al., 2016; Rappaport and Waldman, 2018). Accessibility of *TIMP3* and *CYP7B1* was significantly increased in LSL hens at week 19 on the P+ diet compared to P-, whereas *JAM3*, *ERBB4*, and *DUSP6* were more accessible in LB hens at week 24 on the P- diet compared to P+. The metalloproteinase inhibitor encoded by *TIMP3* regulates ECM remodelling (Hrabia et al., 2022) and an extracellular matrix-bound metalloprotease with anti-inflammatory and anti-angiogenic properties (Mavilio et al., 2016). Changes in *TIMP3* may indicate shifts in gut barrier maintenance or tissue remodelling, which is critical for nutrient uptake (Vilardi et al., 2024). In bile acid metabolism, *CYP7B1* plays a central role (Wu et al., 2023). Alterations could affect lipid digestion and the uptake of intestinal cholesterol (Wu et al., 2023). Tight junction formation and maintaining epithelial barrier function depend on *JAM3* (Schneeberger and Lynch, 2004). Altered accessibility in P- may reflect shifts in paracellular permeability (Itoh et al., 2001). The growth factor receptor *ERBB4* is involved in cellular proliferation and differentiation (Yarden and Sliwkowski, 2001). Negatively regulation of MAPK signalling is mediated by *DUSP6* (Ekerot et al., 2008), a pathway essential for stress response and cell turnover (Yue and López, 2020).

Based on effect of phosphorus-related intestinal adaptation, the results indicate that LB's greater accessibility at innate/lectin programs and LSL's bias toward adaptive signalling set distinct immune-transport baselines that tune barrier and transport when dietary P fluctuates. At laying onset, endocrine cues (estrogens and calcitriol) acting via epigenetic mechanisms likely drive regulatory rewiring and expression changes, reshaping mucosal immune programs and lipid/steroid-metabolism loci and shifting the epithelium from proliferation-biased to transport-optimised. Under P-, LB at week 19 activates a high-absorption program (*CALB1, GUCY2C,* and *GRAMD1C*); LSL responds via ECM/bile-acid remodelling (*TIMP3* and *CYP7B1*) and later (week 24) via Ca^2+^-signalling modules (*SLC8A1* and *RYR1*). At week 24, LB increases accessibility at tight-junction and MAPK-feedback genes (*JAM3* and *DUSP6*). Wnt/TGF-β and actin–cytoskeleton pathways scaffold junctional control, while PPARα/Sp1 link lipid state to transporter turnover. Thus, age and genetics chiefly architect jejunal regulation, with short-term P supply superimposing focused, physiologically meaningful adjustments.

To elucidate overarching functional relationships among the 1778 genes co-regulated at chromatin and transcriptional levels, WGCNA was performed to test whether this set partitions into discrete regulatory modules associated with P related physiological traits. Two major modules with opposite associations to growth and P handling. The purple module, positively correlated with feed intake, body weight, and P in excreta but negatively with P in plasma and jejunal digesta, was enriched for nitrogen (*CA2* and *CA3B*) and lipid metabolism (*ENPP6* and *CHPT1*) and vitamin digestion/absorption (*CUBN* and *SCARB1*) which are known to be essential for lipid and vitamin absorption in the jejunum of avian (Kozyraki et al., 2022; Toomey et al., 2017). This pattern is consistent with enhanced digestive and absorptive capacity; greater intake and nutrient processing reduce digesta P and maintain lower circulating P via rapid utilization and turnover, while absolute P excretion rises in parallel with intake. P is efficiently bound from the circulation and stored in bone and muscle, which is a marker of rapid, efficient growth (Penido and Alon, 2012). Black module (high-flux/redox support), showing a similar correlation pattern and enrichment for central-carbon and PPP enzymes (*MDH2*, *GPI*, *TKT*, *PGD*, and *DLD*) together with glutathione/thioredoxin genes (*MGST1*, *GSTK1*, and *TXNDC12*), likely supports this high-flux state by bolstering epithelial redox balance during elevated transport and metabolic activity. Turquoise module (proliferation/repair state), with strong enrichment for cell-cycle and p53 signalling (e.g*., CDK1/6*, *CCNB1/2*, *CDC20*, *PLK1*; *CCNG1*, *GTSE1*, and *RRM2*) matches its negative correlations with body weight and feed intake and the trend toward higher digesta/plasma P. This suggests a shift toward epithelial renewal/repair at the expense of absorptive specialization, yielding lower growth and reduced transcellular P uptake. Modules red and yellow were also positively associated with body weight and were enriched for core metabolic pathways (amino acid, lipid, carbohydrate, and energy), with the red module additionally tracking higher plasma and digesta P. These subnetworks may represent parallel metabolic routes that support growth while fine-tuning P availability in the lumen and circulation. Together, WGCNA demonstrates that DEG-DAR overlap genes exhibit stronger within-module connectivity and map to functionally coherent networks that differentially relate to P handling and growth, thereby revealing the regulatory architecture underlying the observed phenotypes.

## 5 Conclusion

The results suggest that the genetic background and age of laying hens shape chromatin accessibility and transcriptional landscapes in the jejunum during the transition to egg-laying. Despite subtle effects of dietary phosphorus, chromatin changes reveal strain- and age-specific responses to P-deficient (P-) or supplemented (P+) diet. The critical period between 19- and 24-week highlights strain-specific adaptations to optimize phosphorus utilization. By integrating ATAC-Seq and RNA-Seq, this study uncovered regulatory layers governing intestinal mineral absorption and homeostasis and linked them to P-related physiological traits. These findings provide a foundation for future studies of epigenetic–genetic interactions in mineral metabolism in laying hens.

## Credit Author Statement

**Yosef A. Abitew:** Writing – review & editing, Writing – original draft, Software, Data curation. **Henry Reyer:** Writing – review & editing, Writing – original draft, Data curation. **Frieder Hadlich:** Writing – review & editing, Software, Data curation. **Michael Oster:** Writing – review & editing, Data curation. **Nares Trakooljul:** Writing – review & editing, Data curation. **Vera Sommerfeld:** Writing – review & editing, Resources, Data curation. **Markus Rodehutscord:** Writing – review & editing, Resources, Data curation, Conceptualization. **Klaus Wimmers:** Writing – review & editing, Project administration, Conceptualization. **Siriluck Ponsuksili:** Writing – review & editing, Writing – original draft, Supervision, Project administration, Funding acquisition, Conceptualization.

## Availability of data and materials

The datasets generated and/or analysed during the current study are available at the EMBL-EBI database (www.ebi.ac.uk/arrayexpress) via the accession number E-MTAB-14836. The reviewer's share link (https://www.ebi.ac.uk/biostudies/arrayexpress/studies/E-MTAB-14836?key=4613e4e0-1b95-491d-8050-0e1078ec12f2).

## Declaration of competing interest

We declare that we have no financial and personal relationships with other people or organizations that can inappropriately influence our work, and there is no professional or other personal interest of any nature or kind in any product, service and/or company that could be construed as influencing the content of this paper.
