# Supplementary material for: Chromatin accessibility of the jejunum in two high-yielding laying hen strains under divergent mineral phosphorus supply during the transition to egg laying
Source: Anim Nutr. 2026 Jun 8;26:493–509. doi: 10.1016/j.aninu.2026.01.015 (PMC13356744; doi:10.1016/j.aninu.2026.01.015)
Supplement: Multimedia component 1 [file mmc1.docx]

Abbreviations and full names of genes.

| \| Abbreviations \| Full names \| \| --- \| --- \| \| *ADA* \| Adenosine deaminase \| \| *ADAMTS7* \| ADAM metallopeptidase with thrombospondin type 1 motif 7 \| \| *ADAMTS14* \| ADAM metallopeptidase with thrombospondin type 1 motif 14 \| \| *ADAMTS18* \| ADAM metallopeptidase with thrombospondin type 1 motif 18 \| \| *AKR1D1* \| Aldo-keto reductase family 1 member D1 \| \| *AKR1E2* \| Aldo-keto reductase family 1 member E2 \| \| *ALKBH8* \| Alkb homolog 8, tRNA methyltransferase \| \| *ATF3* \| Activating transcription factor 3 \| \| *CA3B* \| Carbonic anhydrase 3B \| \| *CALB1* \| Calbindin 1 \| \| *CCNB1* \| Cyclin B1 \| \| *CCNB2* \| Cyclin B2 \| \| *CCNE2* \| Cyclin E2 \| \| *CCNG1* \| Cyclin G1 \| \| *CD274* \| CD274 molecule (programmed death-ligand 1; PD-L1) \| \| *CDC7* \| Cell division cycle 7 \| \| *CDC20* \| Cell division cycle 20 \| \| *CDK1* \| Cyclin-dependent kinase 1 \| \| *CDK6* \| Cyclin-dependent kinase 6 \| \| *CDKN1B* \| Cyclin-dependent kinase inhibitor 1B \| \| *CEBPA* \| CCAAT enhancer binding protein alpha \| \| *CEMIP* \| Cell migration inducing hyaluronidase 1 \| \| *CHD1W* \| Chromodomain helicase DNA binding protein 1, W-linked \| \| *CHIR3B10* \| Chicken Ig-like receptor 3B10 \| \| *CHST14* \| Carbohydrate sulfotransferase 14 \| \| *CLOCK* \| Clock circadian regulator \| \| *CUL1* \| Cullin 1 \| \| *CYP7B1* \| Cytochrome P450 family 7 subfamily B member 1 \| \| *DBF4* \| DBF4 zinc finger \| \| *DCLK2* \| Doublecortin-like kinase 2 \| \| *DDX11* \| DEAD/H-box helicase 11 \| \| *DECR1* \| 2,4-dienoyl-CoA reductase 1 \| \| *DENND1B* \| DENN domain containing 1B \| \| *DGAT2* \| Diacylglycerol O-acyltransferase 2 \| \| *DGKZ* \| Diacylglycerol kinase zeta \| \| *DKK4* \| Dickkopf WNT signaling pathway inhibitor 4 \| \| *DLD* \| Dihydrolipoamide dehydrogenase \| \| *DUSP6* \| Dual specificity phosphatase 6 \| \| *EDA* \| Ectodysplasin A \| \| *EI24* \| EI24 autophagy associated transmembrane protein \| \| *ENPP4* \| Ectonucleotide pyrophosphatase/phosphodiesterase 4 \| \| *ENPP8* \| Ectonucleotide pyrophosphatase/phosphodiesterase 8 \| \| *ENTPD8L1* \| Ectonucleoside triphosphate diphosphohydrolase 8-like 1 \| \| *EOMES* \| Eomesodermin \| \| *EPHB2* \| EPH receptor B2 \| \| *ERBB4* \| Erb-b2 receptor tyrosine kinase 4 \| \| *ETV7* \| ETS variant transcription factor 7 \| \| *FAM20A* \| FAM20A Golgi associated secretory pathway pseudokinase \| \| *FGFBP1* \| Fibroblast growth factor binding protein 1 \| \| *FGGY* \| FGGY carbohydrate kinase domain containing \| \| *FOXP4* \| Forkhead box P4 \| \| *FUT9* \| Fucosyltransferase 9 \| \| *GATA2* \| GATA binding protein 2 \| \| *GATA3* \| GATA binding protein 3 \| \| *GHITM* \| Growth hormone inducible transmembrane protein \| \| *GPC1* \| Glypican 1 \| \| *GPI* \| Glucose-6-phosphate isomerase \| \| *GRAMD1C* \| GRAM domain containing 1C \| \| *GSK3A* \| Glycogen synthase kinase 3 alpha \| \| *GSTK1* \| Glutathione S-transferase kappa 1 \| \| *GTSE1* \| G2 and S-phase expressed 1 \| \| *GUCY2C* \| Guanylate cyclase 2C \| \| *GUCY2D* \| Guanylate cyclase 2D, retinal \| \| *GYG2* \| Glycogenin 2 \| \| *HCK* \| HCK proto-oncogene, Src family tyrosine kinase \| \| *HKDC1* \| Hexokinase domain containing 1 \| \| *HS3ST* \| Heparan sulfate-glucosamine 3-sulfotransferase \| \| *INHBB* \| Inhibin subunit beta B \| \| *INPP1* \| Inositol polyphosphate-1-phosphatase \| \| *ISX* \| Intestine specific homeobox \| \| *ITPR1* \| Inositol 1,4,5-trisphosphate receptor type 1 \| \| *JAM3* \| Junctional adhesion molecule 3 \| \| *JUND* \| JunD proto-oncogene, AP-1 transcription factor subunit \| \| *KLHL4* \| Kelch-like family member 4 \| \| *LAMA2* \| Laminin subunit alpha 2 \| \| *LEF1* \| Lymphoid enhancer binding factor 1 \| \| *LHX6* \| LIM homeobox 6 \| \| *LPCAT2* \| Lysophosphatidylcholine acyltransferase 2 \| \| *MAFF* \| MAF bZIP transcription factor F \| \| *MAPK* \| Mitogen-activated protein kinase \| \| *MAPK10* \| Mitogen-activated protein kinase 10 \| \| *MCM5* \| Minichromosome maintenance complex component 5 \| \| *MCM6* \| Minichromosome maintenance complex component 6 \| \| *MDH1* \| Malate dehydrogenase 1 \| \| *MDH2* \| Malate dehydrogenase 2 \| \| *MGAT4C* \| MGAT4 family member C \| \| *MGST1* \| Microsomal glutathione S-transferase 1 \| \| *MHCY8* \| Major histocompatibility complex Y locus 8 \| \| *MHCY58* \| Major histocompatibility complex Y locus 58 \| \| *MMP1* \| Matrix metallopeptidase 1 \| \| *MOXD1* \| Monooxygenase DBH like 1 \| \| *MTMR1* \| Myotubularin related protein 1 \| \| *MYC* \| MYC proto-oncogene, bHLH transcription factor \| \| *NOXA1* \| NADPH oxidase activator 1 \| \| *NRP1* \| Neuropilin 1 \| \| *ORC6* \| Origin recognition complex subunit 6 \| \| *PALD1* \| Phosphatase domain containing paladin 1 \| \| *PAPSS* \| 3′-Phosphoadenosine 5′-phosphosulfate synthase \| \| *PAX5* \| Paired box 5 \| \| *PBX1* \| PBX homeobox 1 \| \| *PHAX* \| Phosphorylated adaptor for RNA export \| \| *PHF20* \| PHD finger protein 20 \| \| *PIK3CD* \| Phosphatidylinositol-4,5-bisphosphate 3-kinase catalytic subunit delta \| \| *PIP4K2A* \| Phosphatidylinositol-5-phosphate 4-kinase type 2 alpha \| \| *PKDCC* \| Protein kinase domain containing, cytoplasmic \| \| *PLA2G2A* \| Phospholipase A2 group IIA \| \| *PLCG1* \| Phospholipase C gamma 1 \| \| *PLK1* \| Polo-like kinase 1 \| \| *PMP22* \| Peripheral myelin protein 22 \| \| *PPARA* \| Peroxisome proliferator-activated receptor alpha \| \| *PPARG* \| Peroxisome proliferator-activated receptor gamma \| \| *PPP1R3* \| Protein phosphatase 1 regulatory subunit 3 \| \| *PPP3CA* \| Protein phosphatase 3 catalytic subunit alpha \| \| *PREP* \| Prolyl endopeptidase \| \| *PRKCB* \| Protein kinase C beta \| \| *PTTG2* \| Pituitary tumor-transforming 2 \| \| *RAPGEFL1* \| Rap guanine nucleotide exchange factor like 1 \| \| *RECQL4* \| RecQ-like helicase 4 \| \| *REXO5* \| RNA exonuclease 5 \| \| *RGN* \| Regucalcin \| \| *RRM2* \| Ribonucleotide reductase regulatory subunit M2 \| \| *RUNX2* \| Runt-related transcription factor 2 \| \| *RUNX3* \| Runt-related transcription factor 3 \| \| *RXRG* \| Retinoid X receptor gamma \| \| *RYR1* \| Ryanodine receptor 1 \| \| *SAAL1* \| Serum amyloid A-like 1 \| \| *SGO1* \| Shugoshin 1 \| \| *SH3BGRL* \| SH3 domain binding glutamate-rich protein-like \| \| *SKP1* \| S-phase kinase associated protein 1 \| \| *SLC8A1* \| Solute carrier family 8 member A1 \| \| *SLC9A2* \| Solute carrier family 9 member A2 \| \| *SLC13A2* \| Solute carrier family 13 member 2 \| \| *SLC24A2* \| Solute carrier family 24 member 2 \| \| *SLC25A24* \| Solute carrier family 25 member 24 \| \| *SLC26A2* \| Solute carrier family 26 member 2 \| \| *SLC30A10* \| Solute carrier family 30 member 10 \| \| *SMAD* \| Mothers against decapentaplegic homolog \| \| *SP1* \| Sp1 transcription factor \| \| *SPIN1* \| Spindlin 1 \| \| *STAT3* \| Signal transducer and activator of transcription 3 \| \| *SYK* \| Spleen-associated tyrosine kinase \| \| *TAP1* \| Transporter 1, ATP-binding cassette subfamily B member \| \| *TASOR2* \| Transcription activation suppressor family member 2 \| \| *TGFB1* \| Transforming growth factor beta 1 \| \| *TGFB3* \| Transforming growth factor beta 3 \| \| *TIMP3* \| Tissue inhibitor of metalloproteinase 3 \| \| *TKT* \| Transketolase \| \| *TLN2* \| Talin 2 \| \| *TNFRSF18* \| TNF receptor superfamily member 18 \| \| *TPH2* \| Tryptophan hydroxylase 2 \| \| *TRIM54* \| Tripartite motif containing 54 \| \| *TSPAN4* \| Tetraspanin 4 \| \| *TXNDC12* \| Thioredoxin domain containing 12 \| \| *UCP3* \| Uncoupling protein 3 \| \| *UNC13D* \| Unc-13 homolog D \| \| *UPK1B* \| Uroplakin 1B \| \| *UTS2R* \| Urotensin 2 receptor \| \| *VANGL1* \| VANGL planar cell polarity protein 1 \| \| *YLEC8* \| C-type lectin-like 8 \| \| *YLEC9* \| C-type lectin-like 9 \| \| *YLEC13* \| C-type lectin-like 13 \| \| *YLEC18* \| C-type lectin-like 18 \|   Table S1 Ingredients and nutrient levels of the experimental diets (dry matter basis, g/kg) (Sommerfeld et al., 2024). | | | | | | |  |
| --- | --- | --- | --- | --- | --- | --- | --- | --- | --- | --- | --- | --- | --- | --- | --- | --- | --- | --- | --- | --- | --- | --- | --- | --- | --- | --- | --- | --- | --- | --- | --- | --- | --- | --- | --- | --- | --- | --- | --- | --- | --- | --- | --- | --- | --- | --- | --- | --- | --- | --- | --- | --- | --- | --- | --- | --- | --- | --- | --- | --- | --- | --- | --- | --- | --- | --- | --- | --- | --- | --- | --- | --- | --- | --- | --- | --- | --- | --- | --- | --- | --- | --- | --- | --- | --- | --- | --- | --- | --- | --- | --- | --- | --- | --- | --- | --- | --- | --- | --- | --- | --- | --- | --- | --- | --- | --- | --- | --- | --- | --- | --- | --- | --- | --- | --- | --- | --- | --- | --- | --- | --- | --- | --- | --- | --- | --- | --- | --- | --- | --- | --- | --- | --- | --- | --- | --- | --- | --- | --- | --- | --- | --- | --- | --- | --- | --- | --- | --- | --- | --- | --- | --- | --- | --- | --- | --- | --- | --- | --- | --- | --- | --- | --- | --- | --- | --- | --- | --- | --- | --- | --- | --- | --- | --- | --- | --- | --- | --- | --- | --- | --- | --- | --- | --- | --- | --- | --- | --- | --- | --- | --- | --- | --- | --- | --- | --- | --- | --- | --- | --- | --- | --- | --- | --- | --- | --- | --- | --- | --- | --- | --- | --- | --- | --- | --- | --- | --- | --- | --- | --- | --- | --- | --- | --- | --- | --- | --- | --- | --- | --- | --- | --- | --- | --- | --- | --- | --- | --- | --- | --- | --- | --- | --- | --- | --- | --- | --- | --- | --- | --- | --- | --- | --- | --- | --- | --- | --- | --- | --- | --- | --- | --- | --- | --- | --- | --- | --- | --- | --- | --- | --- | --- | --- | --- | --- | --- | --- | --- | --- | --- | --- | --- | --- | --- | --- | --- | --- | --- | --- | --- | --- | --- | --- | --- | --- | --- | --- | --- | --- | --- | --- | --- | --- | --- | --- | --- | --- | --- | --- | --- | --- | --- | --- | --- | --- | --- | --- | --- | --- | --- | --- | --- | --- | --- | --- | --- | --- | --- | --- | --- | --- | --- | --- |
| Items | **Developer**  **(weeks 15-16)** | | **Pre-layer**  **(weeks 16-17)** | | **Layer**  **(weeks 17-19; weeks 20-24)** | | |
|  | **P-^1^** | **P+^1^** | **P-** | **P+** | **P-** | **P+** | |
| **Ingredients** |  |  |  |  |  |  | |
| Corn | 619.5 | 617.1 | 589.8 | 585.8 | 599.2 | 595.9 | |
| Soybean meal | 220.0 | 220.0 | 275.5 | 276.2 | 260 | 260 | |
| Alfalfa meal | 120.0 | 120.0 | 60.0 | 60.0 | 30.0 | 30.0 | |
| Soybean oil | 8.0 | 8.0 | 10.0 | 10.0 | 15.0 | 15.0 | |
| DL-Methionine | 1.5 | 1.5 | 3.7 | 3.7 | 4.5 | 4.5 | |
| L-Lysine sulphate | 0.0 | 0.0 | 0.0 | 0.0 | 0.7 | 0.7 | |
| Monocalcium phosphate | 0.0 | 4.7 | 0.0 | 5.0 | 0.0 | 5.0 | |
| Limestone (fine) | 18.2 | 15.9 | 27.5 | 26.8 | 23.1 | 22.4 | |
| Limestone (coarse) | 0.0 | 0.0 | 20.0 | 19.0 | 54.0 | 53.0 | |
| Sodium chloride | 2.3 | 2.3 | 2.8 | 2.8 | 3.0 | 3.0 | |
| Choline chloride | 1.0 | 1.0 | 1.0 | 1.0 | 1.0 | 1.0 | |
| Sodium bicarbonate | 2.0 | 2.0 | 2.2 | 2.2 | 2.0 | 2.0 | |
| Vitamin premix^2^ | 2.0 | 2.0 | 2.0 | 2.0 | 2.0 | 2.0 | |
| Mineral premix^3^ | 0.5 | 0.5 | 0.5 | 0.5 | 0.5 | 0.5 | |
| TiO_2_ | 5.0 | 5.0 | 5.0 | 5.0 | 5.0 | 5.0 | |
| Total | 100.0 | 100.0 | 100.0 | 100.0 | 100.0 | 100.0 | |
| **Nutrients** |  |  |  |  |  |  | |
| P | 3.0 | 4.0 | 3.3 | 4.3 | 3.1 | 4.1 | |
| NPP | 1.3 | 2.3 | 1.4 | 2.4 | 1.3 | 2.3 | |
| Ca | 9.0 | 9.0 | 22.5 | 22.5 | 35.0 | 35.0 | |
| CP | 155 | 155 | 176 | 176 | 168 | 168 | |
| ME, MJ/kg | 11.6 | 11.6 | 11.5 | 11.5 | 11.5 | 11.5 | |
| P = phosphorus; NPP = non-phytate phosphorus; Ca = calcium; CP = crude protein; ME = metabolizable energy.  ^1^ P-, without mineral P supplementation; P+, supplemented with 1 g P/kg feed.  ^2^Vitamin premix (MIAVIT GmbH, Essen, Lower Saxony, Germany) provided per kg of the complete diet: vitamin A 10,000 IU, vitamin D_3_ 3000 IU, vitamin E 30 mg, vitamin K_3_ 2.4 mg, biotin 100 mcg, folic acid 1 mg, vitamin B_1_ 3 mg, vitamin B_2_ 6 mg, vitamin B_6_ 6 mg, vitamin B_12_ 30 mcg, nicotinamide 50 mg, calcium-D-pantothenate 14 mg. | | | | | | |  |
| ^3^ Mineral premix (GELAMIN Gesellschaft für Tierernährung mbH, Memmingen, Bayern, Germany) provided per kg of complete diet: manganese from manganese-(II)-oxide 80 mg, zinc from zinc sulfate monohydrate 60 mg, iron from ferrous-(II)-sulfate monohydrate 25 mg, copper from cupric-(II)-sulfate pentahydrate 7.5 mg, iodine from calcium iodate 0.6 mg, selenium from sodium selenite 0.2 mg. | | | | | | |  |
